# Extract from a Letter to a New England Physician

**Published:** 1862-05

**Authors:** 


### EXTRACT FROM A LETTER TO A NEW ENGLAND PHYSICIAN.

We are in receipt of a letter from a distinguished physician in one of our Western cities, to a brother in Massachusetts, and have permission to make the following extract:

"I am now very constantly employed, and if a moment's leisure is afforded me, there is always something important for me to learn; I have no more time to waste. I look back upon my professional life when associated with you in the practice of medicine in New England, and most heartily regret my inattention and laziness. Like a fool, I thought I knew it about all, and came near spending my life and dying at last, without finding out that I really knew nothing of the business which I had followed. I had been a complete quack, giving medicines which I did not understand, for real or supposed diseases of which I knew very little or nothing. Yes, a very quack, the which, I despise, of all the things which God has made, the most; not wishing however to impute my sin or ignorance, or folly, to Him who made me.

When you propose hereafter to advise medicine, first consider if any is really necessary, and how little will answer the indications, and then select those articles which are comparatively unobjectionable or less injurious than the disease itself, which by doing you can greatly diminish your medicine bills, and at the same time the 'bills of mortality.' I do not mean you, in particular, but all doctors, myself among the number. I used to kill patients in pneumonia with bleeding, blistering, antimony and calomel. I now give Dovers' powder. My patients recover sooner, more certainly, and with vastly greater comfort. Dovers' powder, quinine and whisky, are now the only remedies, and most patients would recover perfectly well without either. I had never studied disease unmodified by treatment. I had never had any such opportunity, for every one sick, was fed on drugs, not a single case was ever found for learning the natural history of disease, within the limits of New England. Pneumonia and pleurisy were fashionably treated by bleeding, purging, blistering, calomel, antimony and opium. 'High fever,' as it was often called, was treated upon 'general principles,' by depletion, whatever might have been its nature or cause. In apoplexy bleeding was universal, and many a poor, worn out, old man or woman, with just enough of life to move about, was bled for some prickling sensations or numbness of the feet or hands; severe pain in the head or elsewhere demanded depletion; after falls and injuries of all sorts, broken bones, sprains, &c., evaporating lotions were preceded by venesection. We were more ignorant than we were born, doing vastly more harm than good, and receiving the wages of sin, as the rewards of righteousness.

Twenty years hence and our present practices may astonish us as much. The multiplicity of our remedies, and the confidence with which they are urged, as valuable in the different manifestations of disease; the present distrust of the powers of nature, unassisted by drugs, to recover us from diseases, and our readiness to take and advise many of the articles in common use with physicians, I have no doubt will yet become as much a matter of surprise and astonishment as are the greatest absurdities of bygone generations."

